# Synthesis of carboxylated silicon phthalocyanine photosensitive microspheres with controllable etching

**DOI:** 10.1080/15685551.2019.1603695

**Published:** 2019-05-03

**Authors:** Dan-Dan Liu, Yu Wang, Guo-Zhi Han

**Affiliations:** College of Chemistry and Molecular Engineering, Nanjing Tech University, Nanjing, China

**Keywords:** Silicon phthalocyanine, photosensitive microsphere, carboxylated, controllable etching

## Abstract

Using a new kind of alkenyl silicon phthalocyanine, styrene (St) and methacrylic acid as monomers and potassium persulfate as an initiator, carboxylated silicon phthalocyanine photosensitive microspheres were synthesized by *in-situ* emulsion copolymerization. The structure and morphology of the microspheres were characterized by Fourier transform infrared spectroscopy (FT-IR)and scanning electron microscopy. Fluorescence spectrometer was used to characterize the optical property of the microspheres. The experimental results show that silicon phthalocyanine molecules with flexible unsaturated side chains have good compatibility with styrene. With the presence of carboxyl groups, the as-prepared photosensitive microspheres show high monodispersity and stability. On the basis of this, 9,10-diphenylanthracene (DPA) as singlet oxygen indicator was used to evaluate the photosensitivity of the microspheres. In addition, we report a novel phenomenon that the microspheres can be controllable etched by light irradiation with the presence of DPA in a specific solvent.

## 1. Introduction

Light-initiated chemiluminescent assay (LiCA), which is a homogeneous immunoassay technology, has been widely applied in analytical chemistry, medical rapid diagnosis and fluorescence sensing [1-3]. One of the key materials in LiCA technology is the polymer photosensitive microsphere coated with a specific fluorescent molecule [4–6]. Under the light excitation with a certain wavelength, the singlet oxygen is generated by the interaction between photosensitive microsphere and O_2_, which triggers the subsequent luminescence process [7–10]. Among commonly used photosensitive molecules, due to 18π electron aromatic conjugated structure and tunable excitation wavelength from visible light to near-infrared regions, phthalocyanine dyes have become an important candidate of photosensitive materials for LiCA and photodynamic therapy [11–13]. Generally, the metal-free phthalocyanine compounds have a cavity with a diameter of about 27 nm, which can coordinate with many metal elements or non-metallic element to form a complex compound, for example, copper phthalocyanine, zinc phthalocyanine and silicon phthalocyanine [5,14,15]. The introduction of these central atoms brings better performance to phthalocyanine for optical applications. As a typical non-metallic coordination phthalocyanine, the advantages of silicon phthalocyanine include two aspects. First, organic long chains can be covalently linked to silicon atoms, thereby weakening molecular rigidity of phthalocyanine and enhancing its solubility. Second, compared with metallic phthalocyanine, silicon phthalocyanine has better biocompatibility.

At present, the preparation strategies of the polymer photosensitive microspheres mainly include adsorption, self-assembly and embedding [16,17]. Among them, the photosensitive microspheres formed by the adsorption and the self-assembly method face the challenge of low stability, and the fluorescent molecules are easy to fall off [18]. The embedding method usually has high requirements for the compatibility of polymer microspheres with photosensitive molecules. Moreover, the chemical environment of the polymer microsphere surface has a great influence on the embedding efficiency of photosensitive molecules. Our previous work shows that the efficiency of the embedding method also relies on the nature of the surface groups of the polymer microspheres [19]. Especially, for those fluorescent molecules with large structural rigidity, it is difficult to obtain higher fluorescence efficiency by the embedding method. To solve this problem, the method of polymerization embedding, in which photosensitive molecules are mixed with monomer before polymerization, has become an effective strategy for synthesis of photosensitive microsphere. However, in the polymerization process, the photosensitive molecules are easily leaked from the micelles, thus resulting in the uneven embedding of the photosensitive molecules in microspheres. Hence, copolymerization of photosensitive molecules possessing polymerizable functional groups with another monomer is an ideal route to synthesize photosensitive microspheres. The synthesized microspheres can obtain stable optical properties and uniform distribution of photosensitive substances [20]. Unfortunately, if the photosensitive molecule has a large rigid structure, the polymerization process is easily disturbed along with low monodispersity and sphericity of the photosensitive microspheres owing to poor compatibility of a rigid structure with the general monomer [21]. So, a key point of the copolymerization method for preparation of photosensitive microspheres lies in the molecular structure of photosensitive molecules.

**Figure 1.**
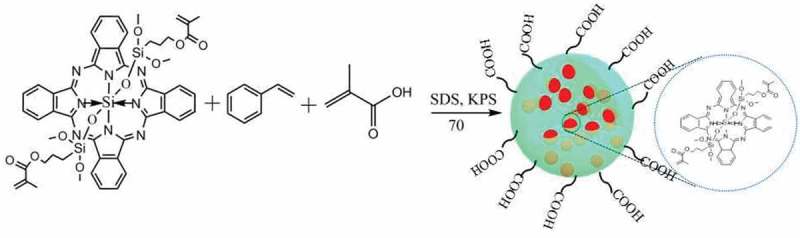
The synthesis route of the photosensitive microsphere.

In this paper, based on our previous work, using alkenyl silicon phthalocyanine, styrene (St) and methacrylic acid (MAA) as monomers and potassium persulfate (KPS) as an initiator, we report a new kind of carboxylated silicon phthalocyanine photosensitive microspheres synthesized by *in-situ* emulsion copolymerization. The experimental results show that silicon phthalocyanine molecules with flexible unsaturated side chains have good compatibility with styrene. With the presence of carboxyl groups, the as-prepared photosensitive microspheres show high monodispersity and stability. The photosensitivity of the microspheres was evaluated by 9,10-diphenylanthracene (DPA), which acts as a singlet oxygen indicator. Interestingly, besides good photosensitivity, a novel phenomenon was observed that the microspheres can be controllable etched by light irradiation in the presence of DPA in a specific solvent. The as-prepared photosensitive microsphere can be applied in controlled etching for fabricating the hierarchical structure. To our best knowledge, there has been no report about this aspect. The synthetic scheme of the photosensitive microsphere is shown in Figure 1.

## 2. Experimental section

### 2.1. Materials and instruments

Styrene (St), MAA, KPS and sodium dodecyl sulfate (SDS) were purchased from Sinopharm Chemical Reagent Co., Ltd. (Shanghai). All reagents were used without further purification. Alkenyl silicon phthalocyanine (SiPc[OSi(C_9_H_17_O_4_)_2_]) was synthesized according to our previous work [22]. High-purity water from a Milli-Q Academic water purification system (Millipore Corp., Billerica, MA, USA) was used in all experiments.

The UV–vis spectra were recorded using a UV-3600 spectrophotometer (Shimadzu, Japan). Scanning electron microscopy (SEM) images were captured using a scanning electron microscope (S-3400, Japan). The FT-IR spectra of studied samples were examined by a Nicolet, AVATAR360 Fourier infrared spectroscopy (Nicolet, USA) in the range of 400–4000 cm^−1^. The fluorescence spectra were recorded on a Cary Eclipse fluorescence spectrometer (Agilent, USA) with laser wavelength at 332 nm. Controllable etching of photosensitive microspheres was validated by an XE-Bio atomic force microscope (AFM) (Park SYSTEMS, Korea).

### 2.2. Synthesis of carboxylated silicon phthalocyanine photosensitive microspheres

To a 250-mL three-necked flask equipped with a thermometer, stirrer and condenser, SiPc[OSi(C_9_H_17_O_4_)_2_] (0.1 g, 0.13 mmol), α-methacrylic acid (0.1 g, 1.16 mmol), styrene (3.7 g, 40 mmol) and 100 mL of deionized water containing 14.0 mg SDS were added. After stirring for 30 min under nitrogen gas at room temperature, the reaction mixture was warmed to 70°C. Subsequently, 1 mL (0.08 g/mL) of KPS solution was quickly added to the system. The reaction was continued for 12 h and then the carboxylated silicon phthalocyanine photosensitive microspheres were collected by centrifugation and re-dispersed in deionized water to form a pale green emulsion.

### 2.3. Photosensitive analysis of carboxylated silicon phthalocyanine photosensitive microspheres

The carboxylated silicon phthalocyanine photosensitive microspheres (0.008 g) were dispersed in 60 mL of deionized water. The solution was divided into four portions on average, and then, 1.0 mL DPA solution (4.80 mmol/L, in N,N-dimethylformamide (DMF)) was added to the above two parts of the solution, respectively, and mixed to form a uniform solution. The remaining two portions were acted as reference solutions. Using laser with a wavelength of 640 nm as the excitation source, UV-vis spectra of the microsphere solutions were recorded under illumination and non-light conditions, respectively. Furthermore, to study the effect of solvent on the photosensitivity of microspheres, the carboxylated silicon phthalocyanine photosensitive microspheres (0.008 g) were dispersed in 60 mL of DMF solvent, and the same experiments were conducted.

## 3. Results and discussion

### 3.1. Structure characterization of the photosensitive microsphere

**Figure 2.**
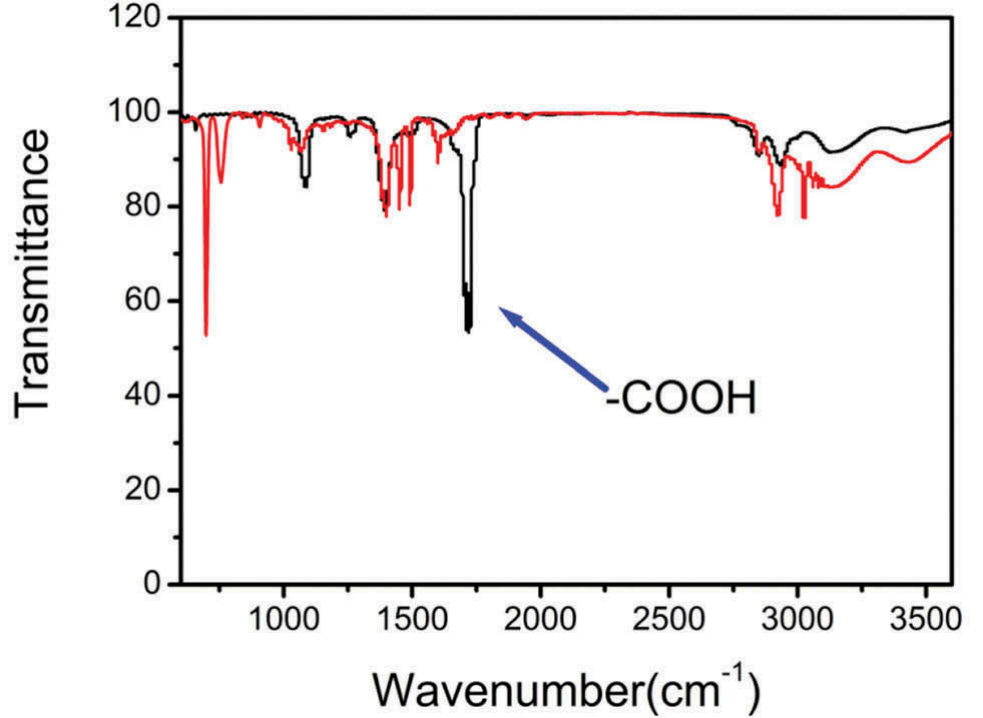
The FT-IR spectra of the as-prepared photosensitive microsphere.

First, FT-IR spectra were used to study the chemical feature of the as-prepared photosensitive microspheres. Figure 2 illustrates the infrared spectra of the carboxylated microspheres and non-carboxylated microspheres. For carboxylated photosensitive microspheres, there is a characteristic absorption peak at 830 cm^−1^, which is attributed to the stretching vibration of the Si-O bond in Si-OH. Two absorption peaks at 580 and 1100 cm^−1^ belong to the bending and stretching vibration of -Si-O-Si-, respectively. The strong absorption peak at 1600 cm^−1^ is attributed to the stretching vibration of C = C bond, while the strong absorption peak at 1723 cm^−1^ is assigned to the stretching vibration of -C = O in the carboxyl group. The absorption peaks at 3080 and 3030 cm^−1^ are attributable to the stretching vibration of the C-H bond in the benzene ring. The absorption peaks at 760 and 690 cm^−1^ are attributed to the bending vibration of the phenyl ring. The above experimental results show that the synthetic route results are credible.

### 3.2. Optimization of synthesis condition

#### 3.2.1. Impact of synthetic temperature on morphology and property

**Figure 3.**
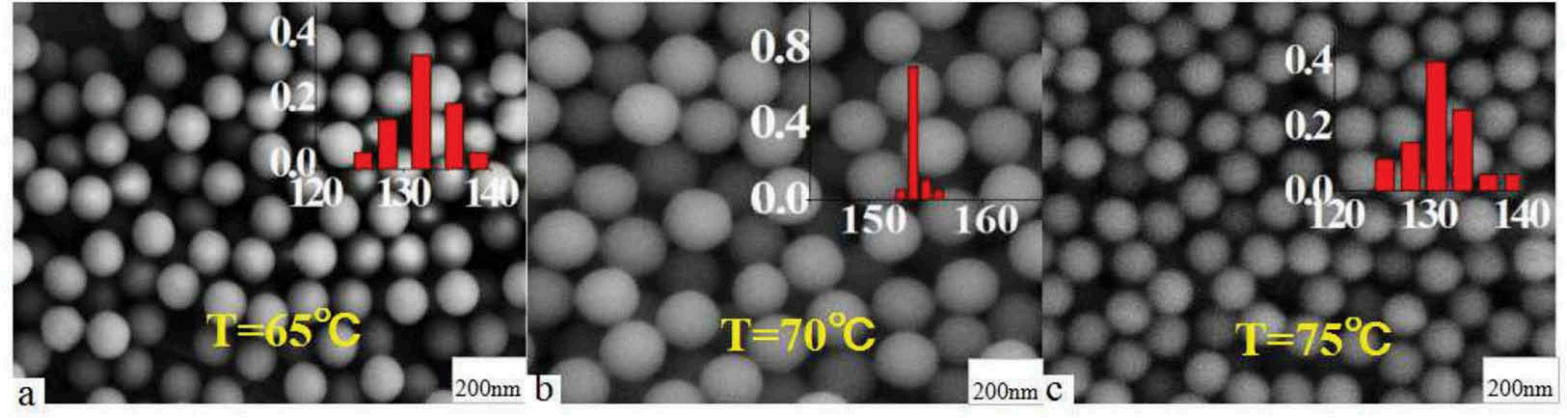
SEM images of carboxylated photosensitive microspheres prepared at different temperatures (styrene: 8 mL, fluorescent dyes: 0.008 g, MAA: 100 μL).

First, the effects of synthetic temperature on the morphology and optical property of the photosensitive microspheres were studied. Figure 3 shows SEM maps of carboxylated photosensitive microspheres synthesized at different temperatures. The experimental results show that the monodispersity and particle size of the carboxylated photosensitive microspheres increase along with the synthetic temperature. When the synthetic temperature is raised from 65°C to 70°C, the average particle size of the microspheres increased from 133 to 144 nm accompanied with an increase of the monodispersity. However, when the temperature exceeded 75°C, the average particle size of the microspheres decreased to about 128 nm, and the distribution of the particle size became wider. The reason may be due to that, as the polymerization temperature increases, the polymer chain length simultaneously increased with the polymerization rate, which facilitates the dispersion of the rigid and bulky silicon phthalocyanine molecules inside the micelles. However, if the temperature is beyond a certain temperature, the thermodynamic stability of the micelles is drastically lowered, which subsequently resulted in a decrease of monodispersity of the polymer microspheres.

**Figure 4.**
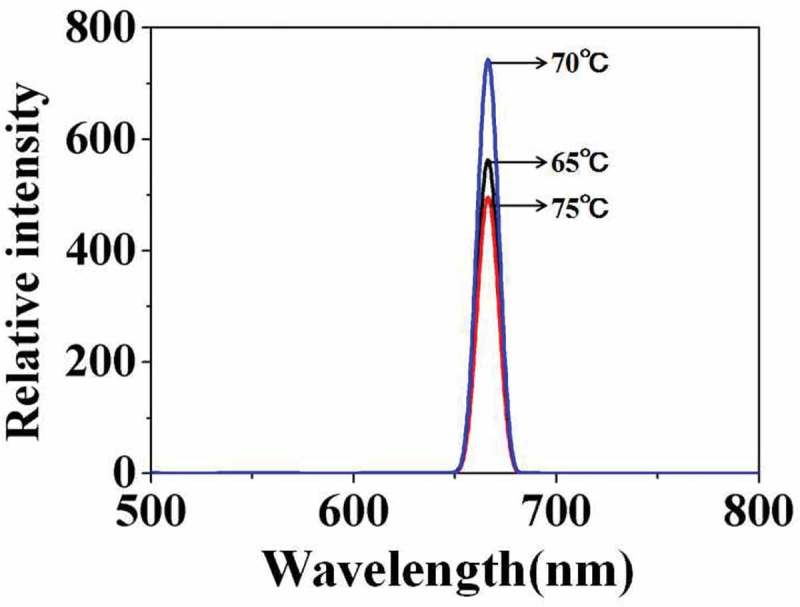
Fluorescence emission spectra of carboxylated photosensitive microspheres prepared at different temperatures.

Figure 4 illustrates the fluorescence emission spectra of carboxylated photosensitive microspheres synthesized at different temperatures. With the temperature increased from 65°C to 70°C, the fluorescence intensity increases from 580 to 750. But when the temperature continued to rise to 75°C, the fluorescence intensity decreased to 500. The results were consistent with the above analysis.

#### 3.2.2. Impact of the amount of fluorescent monomer on carboxylated photosensitive microspheres

**Figure 5.**
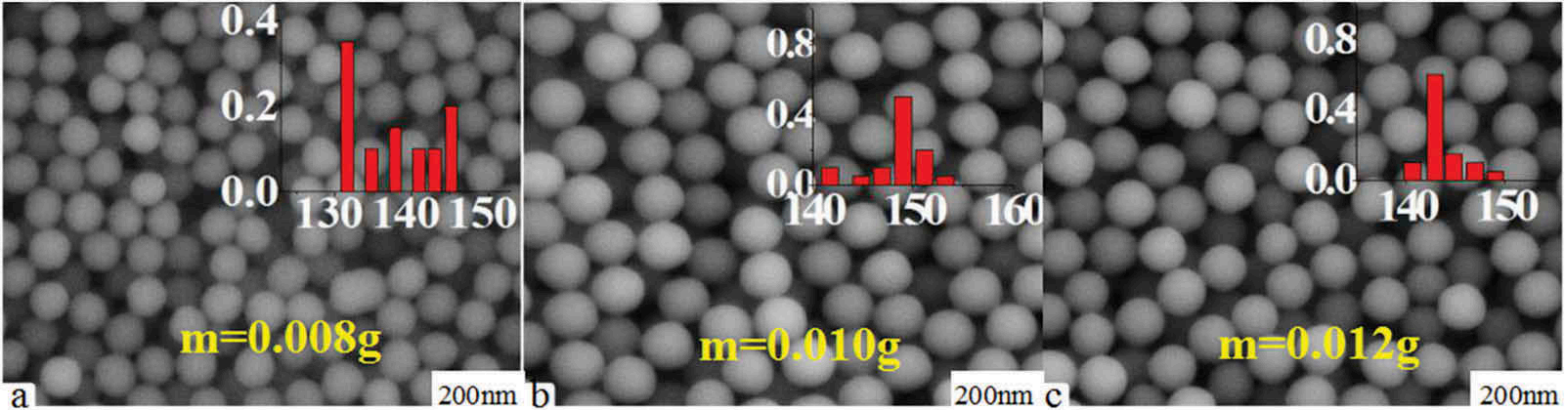
SEM images of carboxylated photosensitive microspheres prepared with different monomer amounts (styrene: 8 mL, temperature: 70 °C, MAA: 100 μL).

Figure 5 shows SEM maps of carboxylated photosensitive microspheres prepared with the different amounts of (SiPc[OSi(C_9_H_17_O_4_)_2_]). Interestingly, the experimental results show that, as the amount of (SiPc[OSi(C_9_H_17_O_4_)_2_]) increased, the particle size of the fluorescent microspheres also presented a trend of increase first and then decrease along with the sphericity and monodispersity. The reason may be attributable to that the volume of the solubilization micelles increased with the increase of the amount of (SiPc[OSi(C_9_H_17_O_4_)_2_]) for its large molecule volume. However, as the amount of (SiPc[OSi(C_9_H_17_O_4_)_2_]) further increases, the thermodynamic stability of the solubilization micelles decreased, subsequently resulted in a decrease of the size and corresponding monodispersity of the microspheres.

**Figure 6.**
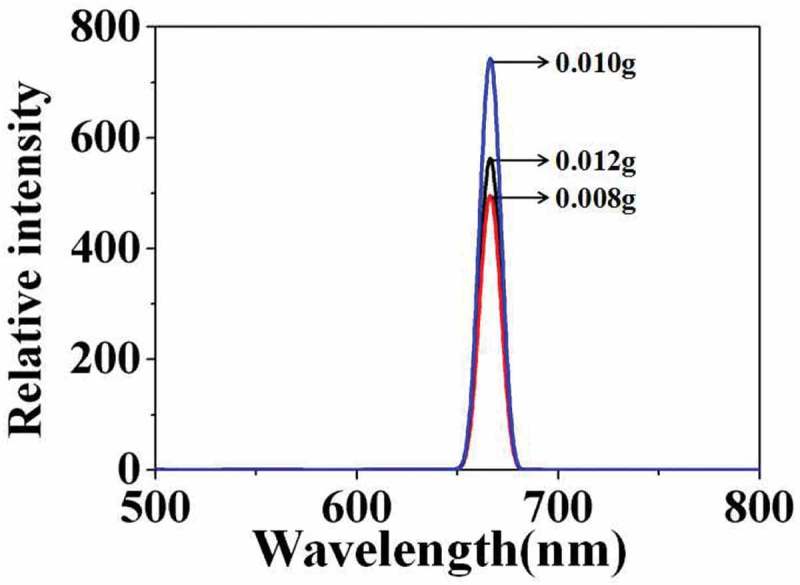
Fluorescence emission spectra of carboxylated photosensitive microspheres prepared with different amounts of (SiPc[OSi(C_9_H_17_O_4_)_2_]) (styrene: 8 mL, temperature: 70°C, MAA: 100 μL).

Figure 6 shows the corresponding fluorescence emission spectra of the above carboxylated photosensitive microspheres. With the amount of (SiPc[OSi(C_9_H_17_O_4_)_2_]) increased from 0.008 to 0.010 g, the fluorescence intensity of the microspheres increases from 500 to 750, whereas reduced to 400 with the amount of (SiPc[OSi(C_9_H_17_O_4_)_2_]) further increased to 0.012 g.

#### 3.2.3. Impact of the amount of MAA on the carboxylated photosensitive microspheres

**Figure 7.**
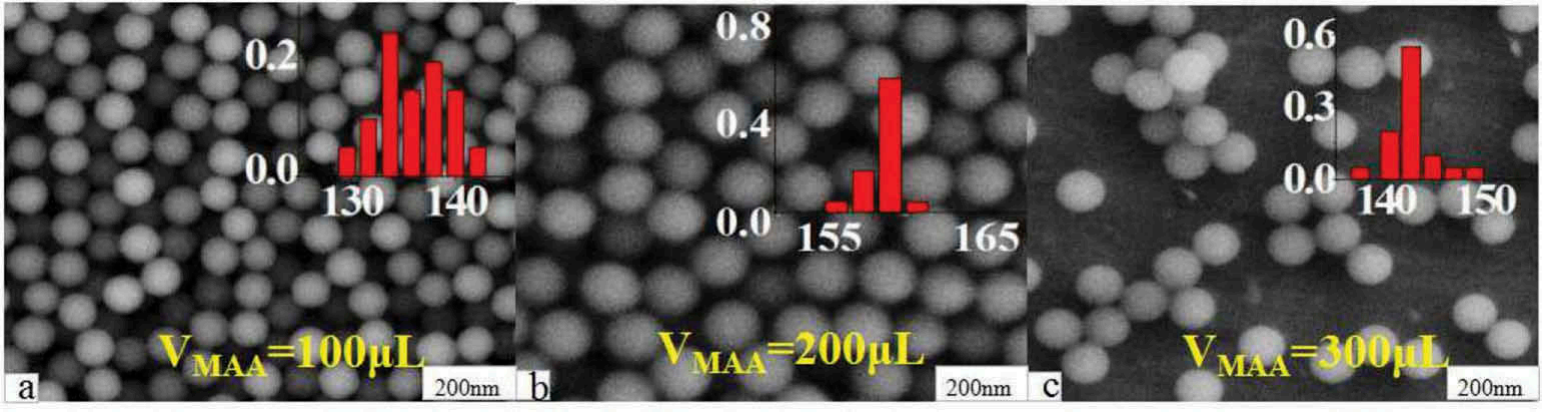
SEM images of carboxylated photosensitive microspheres prepared with different amounts of MAA (temperature: 70°C, fluorescent dyes: 0.01 g, V_St_: 8 mL).

Originating from the coupling of carboxyl groups with biomolecules, carboxylated fluorescence microspheres have become one of the important basic materials in the biomedical and diagnostic field. On the other hand, the carboxyl density (CD) on the surface of the microspheres also has an important effect on the performance of the fluorescent microspheres [22]. Figure 7 shows SEM maps of carboxylated fluorescent microspheres prepared with different amounts of MAA. The experimental results show that, as the amount of MAA increases from 100 to 300 μL, the CD on the surface of the microspheres increased from 2.5 × 10^−3^ to 6.6 × 10^−3^ mmol/mg accompanied with small fluctuations in particle size between 135 and 155 nm.

**Figure 8.**
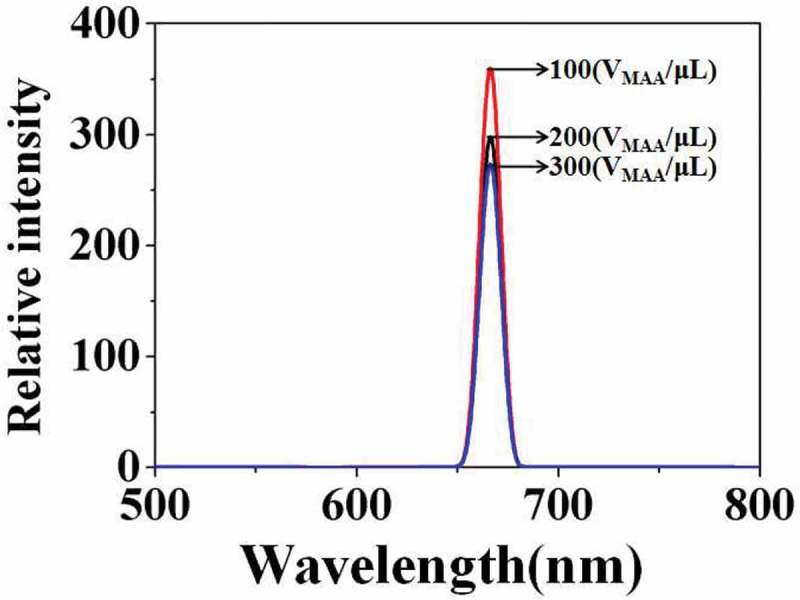
Fluorescence emission spectra of carboxylated photosensitive microspheres prepared with different amounts of MAA.

Figure 8 shows the corresponding fluorescence emission spectra of the above carboxylated photosensitive microspheres prepared with different amounts of MAA. The results show that the change of fluorescence intensity did not coincide with the change of particle size. When the V_MAA_ was increased from 100 to 300 μL, the fluorescence intensity of the photosensitive microspheres was reduced from 370 to 280. The reason may be due to that, as the V_MAA_ increases, the molecule amount of the fluorescent monomer (SiPc[OSi(C_9_H_17_O_4_)_2_]) embedding in the micelle reduced that originating from the increase in polarity on the surface of the microspheres.

### 3.3 Photosensitivity analysis of carboxylated photosensitive microspheres

**Figure 9.**
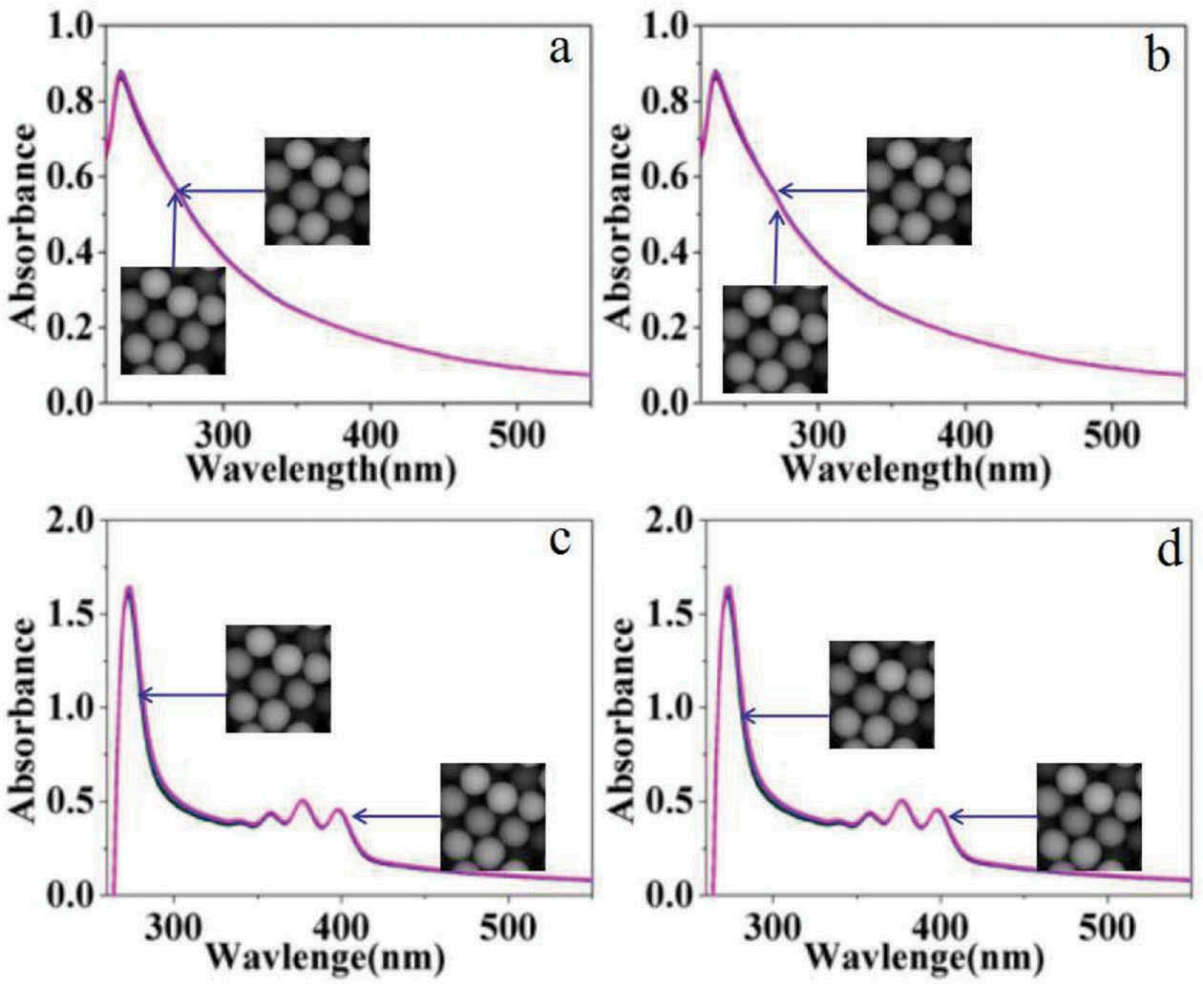
UV-vis spectra of carboxylated photosensitive microspheres under different conditions (H_2_O).

The photosensitivity of carboxylated photosensitive microspheres was studied by UV-vis spectra. First, photosensitive microspheres with a diameter of 162 nm were dispersed in aqueous solution for testing. Among them, Figure 9(a) and (b) shows the changes in the UV-vis spectra of the emulsion without singlet oxygen scavenger of DPA under illumination (640 nm laser) and non-light conditions, respectively. The experimental results show that the UV-vis spectra of the two emulsions almost have no change. Contrastively, Figure 9(c) and (d) shows the changes in the UV-vis spectra of the emulsion in the presence of singlet oxygen scavenger of DPA under illumination (640 nm laser) and non-light conditions, respectively. The characteristic peaks of 350–400 nm are attributed to the characteristic absorption of the aromatic ring in DPA. The UV-vis spectra of the two emulsions also have no obvious change.

**Figure 10.**
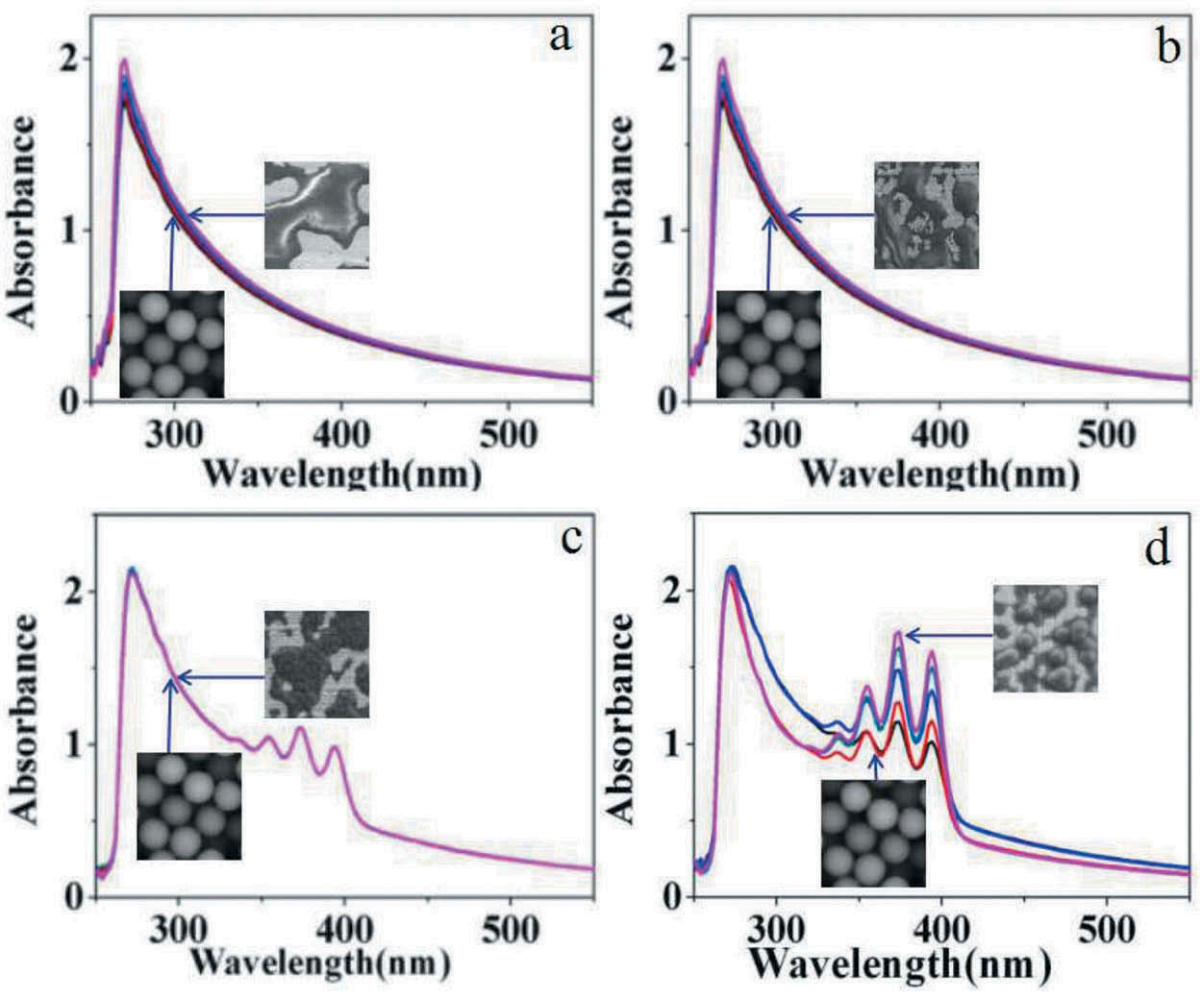
UV-vis spectra of carboxylated photosensitive microspheres in different surroundings (DMF).

However, if the aqueous solvent was replaced by organic DMF, distinct change was observed in UV-vis spectra under the same conditions as shown in Figure 10. Figure 10(a) and (b) shows the UV-vis spectra of the emulsion under illumination (640 nm laser) and non-light conditions, respectively. The experimental results show that the UV-vis spectra of the two emulsions change slightly, which may be caused by the dissolution and corrosion of the carboxylated polymer microspheres by DMF. Figure 10 (c) shows the UV-vis spectra of the emulsion after the addition of a certain amount of DPA solution without illumination, and the intensity of the absorption peak hardly changes with time. But under the illumination of 640 nm laser, the absorption peak intensity is significantly enhanced as shown in Figure 10 (d). According to the above results, we proposed a plausible mechanism. In our inference, DPA molecules adsorbed on the surface of the photosensitive microspheres hinder the dissolution and corrosion of the carboxylated polymer microspheres by DMF. However, with the irradiation of laser, oxygen near the surface of photosensitive microspheres is converted into singlet oxygen which subsequently reacts with DPA on the surface. Then, the DPA molecules dissolved in the solution from the surface of microspheres were enhanced. As a result, the protection of photosensitive microspheres by DPA was decreased, and more polymer was dissolved in DMF. So the intensity of the UV-vis spectra was enhanced along with irradiation of the laser.

Based on the above results, the quantum yield of singlet oxygen of the photosensitive microspheres can be indirectly measured by the following formula:

$$\Phi_{\Delta }=\frac{\left(I_{t}-I_{0}\right)}{I_{0}}$$

where *I*_0_ refers to the maximum absorption value of the DPA characteristic peak before illumination, and *I*_*t*_ refers to the absorption value of the same peak after illumination with a specific time.

**Figure 11.**
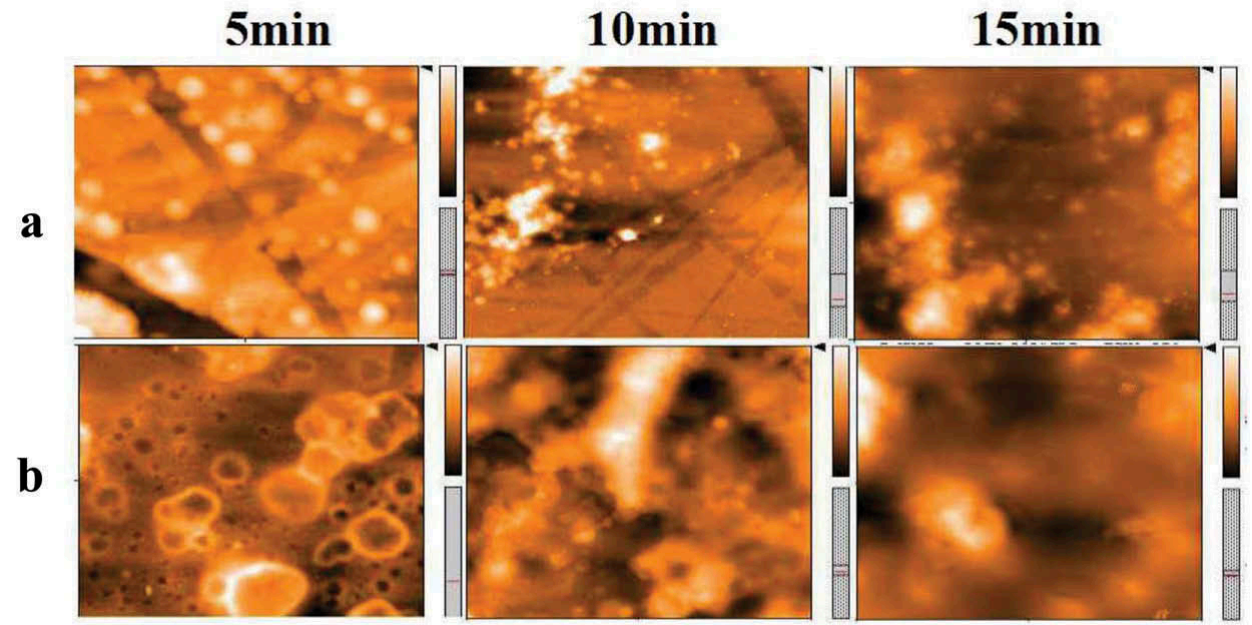
AFM diagram of carboxylated photosensitive microspheres with different conditions.

At last, the above mechanism was further validated by atomic force microscopy as shown in Figure 11. Group A and group B illustrate AFM images of the microsphere DMF solution added with DPA and without DPA under illumination for a different time, respectively. It can be seen from group A that the surface of the microsphere is smooth and flat, whereas the image in group B shows a certain roughness after 5 min. Along with the extension of irradiation time, the surface of microspheres in the absence of DPA becomes much rougher than that in group A. The phenomenon verifies the above theoretical analysis from the side. On the other hand, the experimental results give us a revelation that the system can be applied in controlled etching for fabricating the hierarchical structure.

## 4. Conclusion

In this paper, we report a kind of carboxylated silicon phthalocyanine photosensitive microspheres synthesized by *in-situ* emulsion copolymerization. The structure and morphology of the microspheres were characterized by FT-IR and SEM, and fluorescence spectrometer was used to characterize the optical property of the microspheres. The study shows that silicon phthalocyanine molecules with flexible unsaturated side chains have good compatibility with styrene. With the presence of carboxyl groups, the as-prepared photosensitive microspheres show high monodispersity and stability. Furthermore, DPA as singlet oxygen indicator was used to evaluate the photosensitivity of the microspheres. In addition to good photosensitivity, we report a novel phenomenon that the microspheres can be controllable etched by light irradiation with the presence of DPA in a specific solvent.
